# Activation-induced deaminase expression defines mature B cell lymphoma in the mouse

**DOI:** 10.3389/fimmu.2023.1268930

**Published:** 2023-09-21

**Authors:** Carmen Gómez-Escolar, Ester Marina-Zárate, Almudena R. Ramiro

**Affiliations:** B Lymphocyte Biology Lab, Centro Nacional de Investigaciones Cardiovasculares (CNIC), Madrid, Spain

**Keywords:** λ-MYC, AID, lymphoma, Germinal Center, B lymphocyte

## Abstract

Germinal centers (GCs) are the sites of secondary antibody diversification and underlie the mechanism of action of many vaccination strategies. Activation-induced deaminase (AID) triggers secondary antibody diversification through the introduction of somatic changes in immunoglobulin genes which lead to the generation of antibodies of higher affinity and more specialized effector functions. However, AID can also target other genomic regions, giving rise to mutations and chromosome translocations with oncogenic potential. Many human lymphomas originate from mature B cells that have undergone the GC reaction, such as the diffuse large B cell lymphoma, the follicular lymphoma and Burkitt lymphoma, and carry chromosome translocations. Mature B cell lymphomagenesis has been modeled in the mouse by the genetic introduction of chromosome translocations. Here, we present an in-depth characterization of one such model, λ-MYC mice. We found that young pre-tumor stage mice had a prominent block in early B cell differentiation that resulted in the generation of very aggressive tumors lacking surface B cell receptor (BCR) expression, indicating that a large fraction of tumors in λ-MYC mice arise from B cell precursors rather than from mature B cells. Further, we assessed the contribution of AID to B cell lymphomagenesis in λ-MYC mice by using a genetic tracer of historical AID expression. Only a fraction of tumors contained cells of GC origin as defined by AID expression. AID-experienced tumors associated with longer survival and resembled mature B cell lymphomas. Thus, AID expression defines Burkitt lymphomagenesis in λ-MYC mice.

## 1 Introduction

About 95% of human lymphomas originate from B cells, many of which arise from mature B cells that have undergone the germinal center (GC) reaction. Lymphomas in this category include follicular lymphoma, diffuse large B cell lymphoma, and Burkitt lymphoma (BL) (1). GCs are microanatomical structures formed in secondary lymphoid organs upon B cell stimulation during the immune response and are the sites of secondary antibody (Ab) diversification, which is key to the efficiency of the immune response and underlies the mechanism of action of most vaccination strategies. In GCs, B cells proliferate, modify their immunoglobulin (Ig) genes by somatic hypermutation (SHM), are selected by affinity maturation, and terminally differentiate into memory B cells and plasma cells (2–6). Secondary antibody diversification, which includes SHM and class switch recombination, are initiated by activation-induced deaminase (AID) (7, 8) through the deamination of cytosine residues on Ig genes (9). SHM introduces changes in the variable region of Ig genes, generating related clones of B cells with subtly altered antigen binding capabilities. Those B cells where mutations improve the affinity for the triggering antigen are positively selected in a process called affinity maturation. Class switch recombination is a region-specific recombination reaction that exchanges the primary Cµ constant region encoding IgM by one of the downstream constant regions to give rise to IgG, IgE or IgA. B cells exiting the GC differentiate into high-affinity antibody-secreting plasma cells or memory B cells, which are ready to rapidly respond to re-exposures to the same antigen (10). However, AID can also target other genomic regions, giving rise to mutations (11–13) and chromosome translocations, with major implications for oncogenic transformation (14, 15).

A key regulator in B cell lymphomas is the c-MYC transcription factor, whose deregulated expression is commonly associated with unfavorable prognosis. *c-MYC* is a proto-oncogene that regulates diverse processes such as cell proliferation, cell-cycle progression, the DNA damage response, and apoptosis (16). *c-MYC* was initially identified in BL in the t(8;14) (q24;q32) translocation, which triggers deregulated expression of the c-MYC driven by Ig regulatory elements. *c-Myc-Igh* translocations are dependent on AID activity, being absent in AID-/- B cells (14, 15); in line with this, AID depletion delays lymphoma onset (14, 17). Off-target AID activity is thus crucial for neoplasia formation, establishing a direct link between the GC reaction and B cell lymphomagenesis.

Several transgenic mouse lines have been generated to model pro-tumoral c-MYC overexpression during B cell development under the control of different Ig enhancers. Early on, the Eμ-myc transgenic mouse line was generated by introducing a translocation isolated from a mouse plasmacytoma in which the *c-myc* gene had become coupled to the Ig heavy chain enhancer (18). Eμ-myc mice develop immature and very aggressive B-cell lymphoma and leukemia (19). Later, λ-MYC transgenic mice were developed with a translocated *MYC* gene from a human BL line, placed under the control of Igλ chain regulatory sequences in order to trigger deregulated *c-MYC* expression driven by enhancer elements of the Igλ light chain (20). In the initial description of the model, λ-MYC mice were reported to develop mature B cell lymphomas that share several features with human BL, with a lifespan ranging from 38 to 216 days (20). Subsequently, tumors developed in λ-MYC mice were defined as pre-GC tumors based on the absence of SHM and of the GC-transcriptional regulator *Bcl6* (17). Here, we performed an in-depth characterization of the λ-MYC mouse model, using a genetic tracer to track AID-experienced cells. Young, pre-tumor stage λ-MYC mice showed a prominent block in early B cell differentiation that resulted in the generation of highly aggressive tumors lacking surface BCR expression. Our analysis further showed that the λ-MYC mouse model is heterogeneous, developing at least three types of tumors that differ in aggressiveness and lethality. Only a fraction of the tumors contained AID-experienced cells, which typically represented a minor population within the tumor. Interestingly, mice harboring AID-experienced tumors had higher overall survival, and AID-experienced cells recapitulated the signature of GC and post-GC derived B cell lymphomas. Thus, AID expression associates with the molecular features of mature B cell neoplasia in the mouse.

## 2 Materials and methods

### 2.1 Mice

Male and female mice were used in all experiments. Mice analyzed in this study were at least 4 weeks old and were kept under specific-pathogen-free conditions. All animal procedures conformed to EU Directive 2010/63EU and Recommendation 2007/526/EC regarding the protection of animals used for experimental and other scientific purposes, enacted in Spanish law under RD 53/2013. The procedures were reviewed by the CNIC Institutional Animal Care and Use Committee and approved by the Community of Madrid.

*AicdaCre^+/ki^* mice (15, 21) and *Rosa26tdTom^+/ki^* mice were obtained from the Jackson Laboratory (007770 and 007909) and were crossed to generate *AicdaCre^+/ki^;R26tdTom^+/ki^* mice. λ-MYC mice (*λ-MYC^+/TG^*) (20) were then bred to *AicdaCre^+/ki^;R26tdTom^+/ki^* mice to generate *λ-MYC^+/TG^*;*AicdaCre^+/ki^;R26tdTom^+/ki^* mice, hereafter λ-MYC Tom^+/ki^ mice. For the kinetics experiments, we included only pre-tumor stage mice without signs of disease. For all other experiments, mice were monitored twice a week for lymphoma development until visible signs of disease appeared. Mice were sacrificed when moribund, and the spleen and lymph nodes were collected and analyzed. We define tumor cells as those originated from sick mice harboring severe enlargement of lymph nodes with massive B cell infiltration.

### 2.2 Flow cytometry

Mice were sacrificed, and spleen, lymph nodes, femurs, and tibias were extracted and conserved on ice in PBS supplemented with 2% FBS. Cell suspensions were generated, depleted of erythrocytes by incubation with ACK Lysing Buffer (Lonza, 10-548E), and then incubated with anti-mouse CD16/CD32 antibodies (eBiosciences) to block Fc receptors. Cells were stained with combinations of the following fluorochrome-conjugated antibodies: B220-BV421/FITC (RA3-6B2, BD Horizon), CD19-APCeF780 (ID3, eBiosciences), CD25-APC (PC61.5, Tonbo Biosciences), GL7-FITC (BD GL-7, Pharmingen), GL7-PerCP-CY5.5 (GL-7, Biolegend), IgD-Biotin (11-26, Southern Biotech), IgD-FITC (11-26c.2a, BD Pharmingen), IgM-APC/FITC (IL/41, BD Pharmingen), ST-BV421/PE-CY7 (BD Biosciences) Finally, a viability marker was added to the labeled cell suspension.

Intracellular stainings were performed using the Foxp3/Transcription Factor Staining Buffer Set (eBioscience, 00-5523-00). After FC blocking, cell-surface antigens were stained with selected fluorochrome-conjugated Abs and the viability marker. Cells were additionally fixed with 1% paraformaldehyde for 40 minutes on ice to prevent leakage of Tomato fluorescent proteins. Subsequently, cells were fixed and permeabilized according to the manufacturer’s standard protocol, and stained intracellularly with c-MYC (D84C12, Cell Signaling) or KI67 (SP6, Abcam) and GARAB-AF647 (Life Technologies) secondary antibody. Samples were acquired with LSRFortessa or FACSCanto instruments (BD Biosciences), and data were analyzed with FlowJo V10.4.2 software.

### 2.3 RT-qPCR

Bone marrow (BM) and spleens were harvested from four 8-week-old mice (two λ-MYC and two WT controls), and RNA was extracted from FACS-sorted BM populations (Pre-Pro-B [B220+CD19-], Pro-B [B220+CD19+IgD-IgM-CD25-], Pre-B [B220+CD19+IgD-IgM-CD25+], and immature B cells [B220+CD19+IgD-IgM+CD25-]) and from recirculating splenic B cells obtained by immunomagnetic negative selection with CD43 MACS Microbeads (Miltenyi Biotec-CD43 (Ly-48) Microbeads Mouse). cDNA was synthesized using the High-Capacity cDNA Reverse Transcription Kit (Applied Biosystems) and quantified by SYBR green assay (Applied Biosystems) with normalization to GAPDH expression. The following primers were used: *GAPDH* (forward) 5′-TGA AGC AGG CAT CTG AGG G-3′, (reverse) 5′-CGA AGG TGG AAG AGT GGG AG-3′; mouse *c-Myc* (forward) 5’ GAG CTC CTC GAG CTG TTT GA 3’, (reverse) 5’ AGT TCA CGT TGA GGG GCA TC 3’; human c-MYC (forward) 5’ GGA CCC GCT TCT CTG AAA GG 3’, (reverse) 5’ GAG GCT GCT GGT TTT CCA CTA 3’.

### 2.4 Bulk RNA-sequencing

Two λ-MYC Tom^+/ki^ mice harboring IgM+Tom+ tumors were analyzed by RNA-sequencing. From each mouse, B220+IgM+Tom+ (Tom+) and B220+IgM+Tom- (Tom-) splenocytes were purified using a Sy3200 Cell Sorter (SonyBiotechnology). Total RNA was extracted using the PicoPureTM RNA Isolation Kit (ThermoFisher Scientific), and barcoded RNA-seq libraries were generated using the NEBNext RNA Library preparation kit (Illumina). Library size was measured using the Agilent 2100 Bioanalyzer DNA 1000 chip. Libraries were sequenced with a HiSeq2500 sequencer (Illumina) to generate 60-base single reads. FastQ files for each sample were obtained using CASAVA v1.8 software (Illumina). Sequencing reads were pre-processed with a pipeline that used FastQC (22) to assess read quality and Cutadapt (23) to trim sequencing reads (eliminating Illumina adaptor remains) and to discard reads < 30 bp. The resulting reads were mapped against the mouse transcriptome (GRCm38, release 76) and quantified using Salmon algorithm (24). Data were then processed with a differential expression analysis pipeline that used Bioconductor package EdgeR (25) for normalization and differential expression testing of paired samples with quasi-likelihood F-*test* (QLF). Differentially expressed genes (DEGs) between Tom+ and Tom- were selected based on an adjusted P-value < 0.05. Pathway enrichment analysis was done with Ingenuity Pathway Analysis with DEGs with an absolute FC>1.2. Hierarchical clustering was performed using the average linkage method based on Euclidian distance (Genesis 1.7.7) and the significantly DEGs were identified by ANOVA (26). GSEA was performed with pre-ranked log fold-change values between Tom+ and Tom- lymphoma cells and the gene set PASQUALUCCI_LYMPHOMA_BY_GC_STAGE_DN (17).

### 2.5 Statistical analysis

Data are presented as means ± standard deviation. Statistical analysis was performed in GraphPad Prism (version 8.0.2 for Windows, GraphPad Software, La Jolla California USA, www.graphpad.com). Normality of the data was assessed with the D’Agostino & Pearson normality test. For data with a Gaussian distribution, two-tailed unpaired Student t test was used when comparing two experimental groups and one-way ANOVA when comparing three experimental groups. For survival analysis, Kaplan-Meier curves were generated and compared using the log-rank (Mantel-Cox) test. Differences were considered statistically significant at P < 0.05.

## 3 Results

### 3.1 Abnormal B cell differentiation in the bone marrow of pre-tumor λ-MYC mice

**Figure 1:**
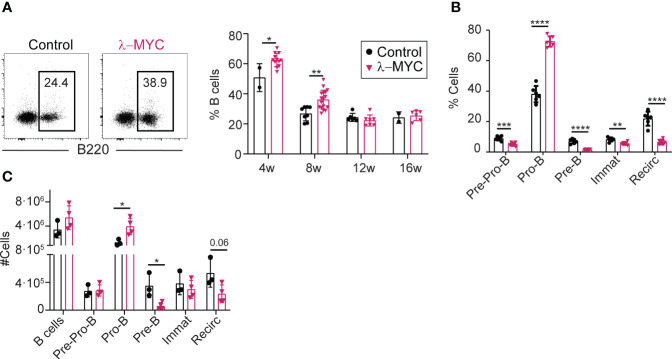
Abnormal B cell differentiation in the bone marrow of pre-tumor stage λ-MYC mice. **(A)** Representative flow cytometry plots (8-week-old mice) and quantification of B cells at different time-points in the BM of control (4w, n = 2; 8w, n = 8; 12w, n = 6; 16w, n = 2) and λ-MYC (4w, n = 12; 8w, n = 17; 12w, n = 7; 16w, n = 6) mice. **(B)** Quantification of B cell (B220+) populations in BM from 8-week-old control (n = 6) and λ-MYC (n = 6) mice: Pre-Pro-B (B220+CD19-), Pro-B (B220+CD19+IgD-IgM-CD25-), Pre-B (B220+CD19+IgD-IgM-CD25+), immature (B220+CD19+IgD-IgM+CD25-), and recirculating (B220+CD19+IgD+). **(C)** Absolute numbers of the B cell subsets shown in panels **(A, B)** from three control and four λ-MYC 8-week-old mice. Data presented as means ± standard deviation and analyzed by unpaired t test. *P<0.05, **P<0.01, ***P<0.001, and ****P<0.0001.

To study the role of AID in MYC-driven lymphomas, we made use of the λ-MYC model, a transgenic mouse model of MYC-driven lymphomagenesis where a translocated *MYC* gene from a human BL line was placed under the control of λ chain regulatory sequences (20). To evaluate the effect of c-MYC overexpression in this mouse model, we first studied B cell development in the bone marrow (BM) of young, pre-tumor stage λ-MYC mice, defined as mice that do not show tumoral masses or signs of disease (**Figure 1**). WT littermates (*λ-MYC^+/+^*) were used as controls (hereafter, controls). At 4-8 weeks of age, λ-MYC mice had a significantly higher proportion of B220+ B cells, but this difference was not seen in older mice (**Figure 1A**). At 8 weeks of age, λ-MYC mice had a markedly elevated proportion of Pro-B cells, whereas all other populations were underrepresented (**Figure 1B**). Very similar differences were found when absolute numbers, rather than proportions, were assessed (**Figure 1C**), indicating that B cell differentiation is severely altered in λ-MYC mice.

**Figure 2:**
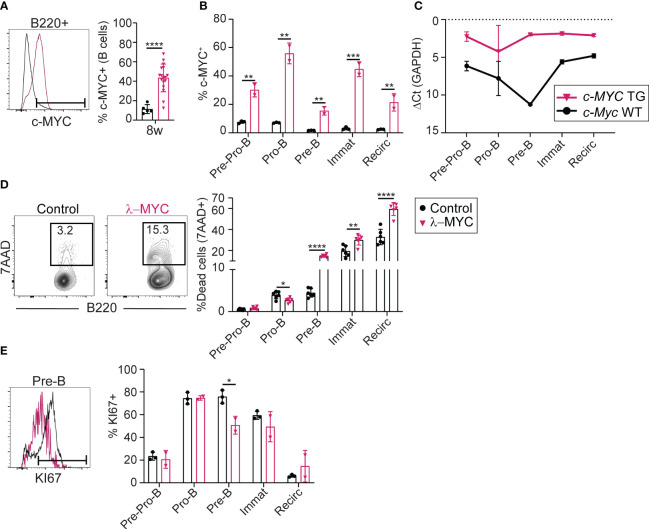
The *c-MYC* transgene is expressed very early in B cell differentiation **(A)** Representative flow cytometry plot and quantification of c-MYC expression in B cells from the BM of 8-week-old control (n = 5) and λ-MYC (n = 17) mice. **(B)** Quantification of c-MYC expression in B cell (B220+) populations from 8-week-old control (n = 3) and λ-MYC mice (n = 2). **(C)** RT-qPCR analysis of endogenous (c-*Myc* WT) and transgenic (c-*MYC* TG) transcription in sorted B cell (B220+) populations (see **Figure 1**) from 8-week-old control (n =2) and λ-MYC mice (n = 2). **(D)** Representative flow cytometry plot and quantification of dead 7AAD+ cells in the BM of 8-week-old control (n = 3) and λ-MYC (n = 2) mice. **(E)** Representative flow cytometry plot and quantification of proliferating Ki-67+ cells in the BM of 8-week-old control (n = 3) and λ-MYC (n = 2) mice. Data presented as means ± standard deviation and analyzed by unpaired t test. *P<0.05, **P<0.01, ***P<0.001, and ****P<0.0001.

To further characterize BM differentiation in pre-tumor stage λ-MYC mice, we analyzed the expression of c-MYC protein and RNA (**Figures 2A-C**). The proportion of c-MYC-expressing B cells was 5-fold higher in λ-MYC BM, as measured by flow cytometry (**Figure 2A**). c-MYC overexpression was detectable as early as the Pre-Pro-B cell stage, and the proportion of c-MYC expressing cells differed among the different B cell differentiation subsets (**Figure 2B**). RT-qPCR analysis detected *c-MYC* transgene expression from the Pre-Pro-B cell stage, with a near constant expression level at all differentiation stages in the BM (**Figure 2C**). In contrast, transcription of endogenous *c-Myc* in control mice showed a transient sharp decline at the Pre-B cell stage (**Figure 2C**). Thus, transgenic *c-MYC* overexpression occurs very early and is sustained in BM B cell precursors of λ-MYC mice, while endogenous c-MYC protein is regulated during B cell differentiation.

We next analyzed cell death and proliferation during early B cell differentiation in pre-tumor stage λ-MYC mice. Pro-B cells from λ-MYC mice showed increased survival compared with controls (**Figure 2D**), but the proportion of proliferating Pro-B cells was unaltered (**Figure 2E**). In contrast, viability and proliferation were both sharply reduced in λ-MYC Pre-B cells (**Figures 2D, E**). B cell survival was also compromised at immature and recirculating B cell subsets (**Figure 2D**) of λ-MYC mice. These data indicate that pre-tumor stage λ-MYC mice have dramatic alterations in B cell differentiation, at least in part due to a strong block at the Pro-B cell stage. This block associates with increased survival of Pro-B cells, together with compromised survival at later developmental stages and reduced Pre-B cell proliferation.

### 3.2 B cell lymphopenia in secondary lymphoid organs of pre-tumor stage λ-MYC mice

**Figure 3:**
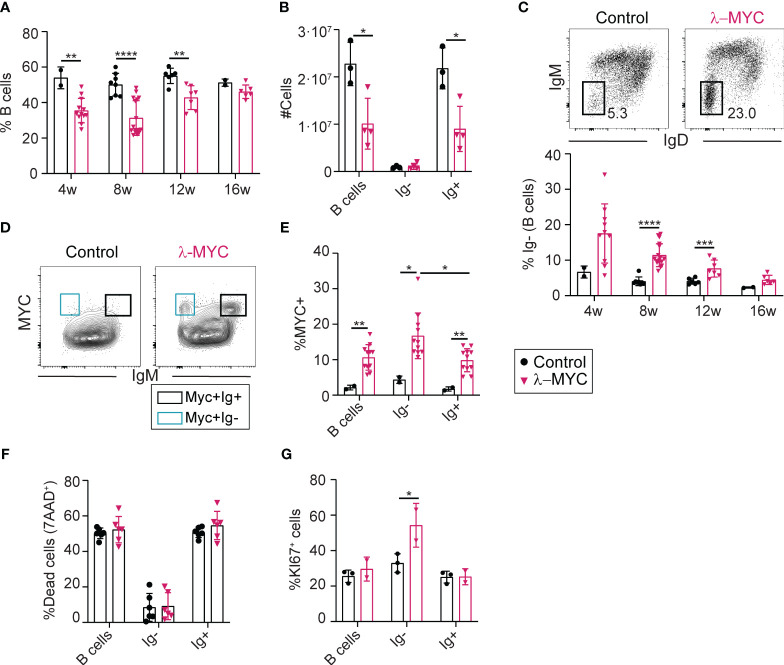
Reduced numbers of B cells in the spleen of λ-MYC mice. **(A)** Quantification of splenic B cells (B220+) in control (4w, n = 2; 8w, n = 8; 12w, n = 6; 16w, n = 2) and λ-MYC (4w, n = 12; 8w, n = 17; 12w, n = 7; 16w, n = 6) mice at the indicated time points. **(B)** Absolute numbers of B cells, IgM-IgD- B cells, and cells expressing IgM and/or IgD in the spleen of 8-week-old control (n = 3) and λ-MYC (n = 4) mice. **(C)** Representative flow cytometry plots and quantification of the proportion of IgM-IgD- B cells (Ig-) in the spleen of control and λ- mice from panel **(A)**. **(D)** Representative flow cytometry plot of IgM and c-MYC expression in B cells of control and λ-MYC mice. **(E)** Quantification of c-MYC+ cells as a percentage within B220+ B cell, Ig- B cell, and Ig+ B cell populations in the spleen of 8-week-old control (n = 2) and λ-MYC (n = 11) mice. **(F)** Quantification of dead cells (7AAD+) in the spleen of 8-week-old control (n = 6) and λ-MYC (n = 6) mice. **(G)** Quantification of proliferating KI67+ cells in the spleen of 8-week-old control (n = 3) and λ-MYC (n = 2) mice. Data presented as means ± standard deviation and analyzed by unpaired t test. *P<0.05, **P<0.01, ***P<0.001, and ****P<0.0001.

We next analyzed the effect of c-MYC overexpression on peripheral B cell populations. The splenic B cell population in pre-tumor stage λ-MYC mice was significantly reduced relative to controls, both as a proportion of the total splenocytes (**Figures 3A**; **S1A**) and in terms of absolute numbers (**Figure 3B**). The lymphopenia in λ-MYC mice was accompanied by a lack of IgM and IgD expression in a considerable proportion of splenic B220+ cells (hereafter, Ig- cells), with the Ig- population being more prominent at 4 weeks of age and decreasing thereafter (**Figures 3C**; **S1B**). In 4-week-old λ-MYC mice, all Ig- splenic B cells expressed either GL7 or CD43 (**Figure S2A**), markers of distinct subsets during early B cell differentiation in the BM (27, 28). These results indicate that early B cell progenitors accumulate in the spleens of young λ-MYC mice.

Flow cytometry analysis of c-MYC expression in peripheral B cells identified two clearly distinct c-MYC+ cell populations in 8-week-old λ-MYC mice that differed in the surface expression of IgM/ (MYC+Ig- and MYC+Ig+), whereas wild type controls were lacking these populations (**Figure 3D**). The c-MYC+ population accounted for about 10% of total B cells in the spleen (**Figure 3E**) and close to 5% in the lymph nodes (**Figure S2B**), with c-MYC expression more prevalent in the Ig- splenic B cell population (**Figures 3E**; **S2B**).

To gain insight into the phenotypic differences between splenic B cells of λ-MYC and control mice, we analyzed cell death and proliferation by flow cytometry (**Figures 3F, G**). In contrast to BM (**Figure 2D**), there were no between-genotype differences in B cell survival in the spleen (**Figure 3F**). However, the proportion of proliferating Ig- cells was significantly higher in λ-MYC mice (**Figure 3G**), consistently with the idea that they contain a population of BM precursors. Together, these data indicate that deregulated c-MYC expression in λ-MYC mice leads to peripheral B cell lymphopenia resulting from defects in B cell generation at two different levels: impaired BM differentiation and aberrant migration of B cell precursors from the BM.

### 3.3 Heterogeneity of tumors developed in λ-MYC mice

To assess the specific role of AID in MYC-driven lymphomagenesis, we combined the λ-MYC lymphoma model with the *AicdaCre^+/ki^* and *R26tdTom^+/ki^* alleles (15, 21) (**Figure S3A**). The resulting *AicdaCre^+/ki^;R26tdTom^+/ki^; λ-Myc^+/TG^* mice (hereafter λ-MYC Tom^+/ki^) allowed genetic tracing of cells that had expressed AID throughout MYC-driven lymphoma generation. As controls, we used *AicdaCre^+/ki^;R26tdTom^+/ki^; λ-Myc^+/+^* littermates (hereafter, controls). Flow cytometry analysis showed the presence of Tom+ cells in the spleen and lymph nodes of pre-tumor stage λ-MYC Tom^+/ki^ mice and littermate controls (**Figure S3B**).

**Figure 4:**
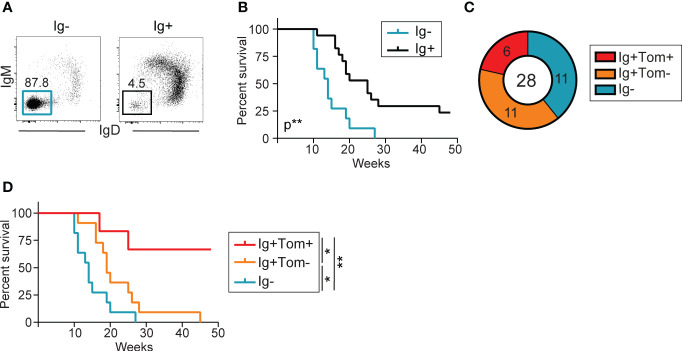
Low incidence of AID-experienced tumors in λ-MYC mice. **(A)** Representative flow cytometry plot of IgM and IgD expression in the splenic B cell populations of two mice, one harboring an Ig- tumor and the other an Ig+ tumor. **(B)** Kaplan-Meier survival curves for λ-MYC Tom+/ki mice harboring Ig- (n = 11) and Ig+ (n = 17) tumors and monitored over 48 weeks. **(C)** Pie chart showing the numbers and proportion of mice harboring IgM- (cyan), Ig+Tom- (orange), and Ig+Tom+ tumors (red) in all λ-MYC Tom^+/ki^ mice analyzed. **(D)** Kaplan-Meier survival curves for λ-MYC Tom+/ki mice harboring tumors from the three groups shown in **(C)** Data were analyzed by log-rank (Mantel-Cox) test. *P ≤ 0.05 and **P<0.01.

Flow cytometry analysis of splenocytes from sick λ-MYC Tom^+/ki^ mice revealed tumor heterogeneity, with one group of animals harboring tumors dominated by Ig- B cells (Ig- tumors), whereas others had tumors containing mostly Ig+ cells (Ig+ tumors) (**Figure 4A**). Ig- tumors presumably originated from early B cell precursors, and these mice had shorter survival, indicating a more aggressive phenotype (**Figure 4B**). Approximately one third of the Ig+ tumors (6/17) contained a fraction of AID-experienced cells, detected by Tomato expression (Ig+Tom+ tumors; >10% Tom+ B cells) (**Figure 4C**). The mice with Ig+Tom+ tumors had better overall survival (**Figure 4D**), suggesting that AID expression is associated with better prognosis in λ-MYC mice.

### 3.4 Tom+ and Tom- lymphoma cells are transcriptionally distinct

**Figure 5:**
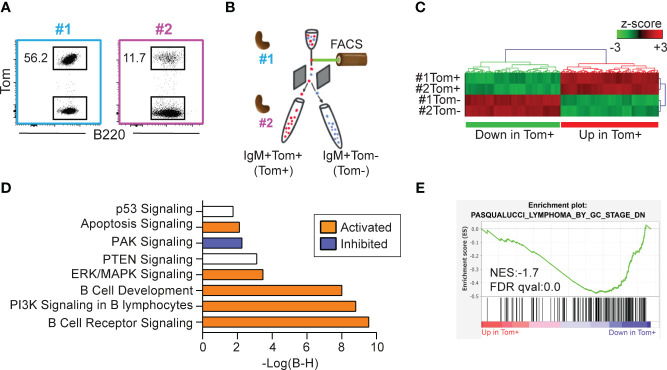
Tom+ and Tom- lymphoma cells are transcriptionally distinct. **(A)** Flow cytometry plots of the two IgM+Tom+ tumors (#1 and #2) selected for RNA-sequencing. The proportion of Tom+ cells within the B cell population is shown. **(B)** Experimental design: IgM+Tom+ (Tom+) and IgM+Tom- (Tom-) cells were isolated by flow cytometry from the spleens of two mice harboring IgM+Tom+ tumors. The four samples were analyzed by RNA-seq. **(C)** Heatmap showing hierarchical clustering of the 177 differentially expressed genes (DEGs) between Tom+ and Tom- cells (ANOVA). Expression values are presented as z-scores. **(D)** Pathway enrichment analysis (IPA) of DEGs between Tom+ and Tom- samples. **(E)** GSEA performed with pre-ranked log fold-change values between Tom+ and Tom- lymphoma cells and the gene set PASQUALUCCI_LYMPHOMA_BY_GC_STAGE_DN (17).

To characterize the impact of AID expression on lymphoma B cells, we performed RNA-sequencing of IgM+Tom+ (Tom+) and IgM+Tom- (Tom-) B lymphoma cells isolated by flow cytometry from two tumors from two independent mice (tumors #1 and #2) (**Figures 5A, B**). Differential gene expression analysis between the Tom+ and Tom- samples was then used as the input for hierarchical clustering (**Figure 5C**) and pathway enrichment analysis (**Figure 5D**) with Ingenuity Pathway Analysis (IPA) software.

Pathways significantly activated in Tom+ cells included the apoptotic signaling pathway and cell death receptor signaling (**Figure 5D**). The phosphatidyl-inositol tri-phosphate (PI3K) signaling pathway—an important signaling pathway activated in BL (29)—was activated in Tom+ cells. In addition, the negative regulator of the PI3K pathway PTEN was significantly deregulated. Other important signaling pathways such as ERK/MAPK were significantly activated in Tom+ cells. Likewise, B cell receptor signaling, and B cell development pathways were active in Tom+ cells. On the other hand, PAK signaling pathway was inhibited in Tom+ lymphoma cells compared to Tom- cells.

To further study the molecular identity of Tom+ and Tom- cells, we performed GSEA analysis using a set of genes that were downregulated in tumors from λ-MYC-IμBCL6 mice, defined as tumors of GC or post-GC origin, compared to tumors from λ-MYC, defined as pre-GC-origin tumors (17). Interestingly, these genes were significantly downregulated in Tom+ cells compared to Tom- cells (**Figure 5E**), indicating that Tom+ cells are transcriptionally similar to GC-derived tumors. Together, these results indicate that AID expression alone determines the transcriptional identity of the tumor cells with a hallmark signature distinctive of mature subtypes of B cell lymphomas.

## 4 Discussion

In this study, we examined the role of AID in MYC-driven lymphomagenesis by generating a reporter model of AID expression in the λ-MYC model of B cell lymphomagenesis. The original λ-MYC model was reported as a mouse model of Burkitt lymphoma in 2000 (20), and was later redefined as a model for pre-GC tumors (17). However, an in-depth characterization of the model was lacking.

Unexpectedly, we found that pre-tumor stage λ-MYC mice showed a marked block at the Pro-B cell stage, accompanied by a pronounced reduction in the subsequent Pre-B cell subset. Examination of secondary lymphoid organs in pre-tumor stage mice revealed B cell lymphopenia, with significantly reduced numbers of total and mature Ig+ B cells in spleen and lymph nodes. This phenotype roughly resembled pre-tumor stage Eμ-myc mice (30). In Eμ-myc mice, *c-myc* gene is coupled to the lymphoid-specific IgH enhancer Eμ. Since this lymphoid-specific enhancer is responsible for Cμ transcriptional activity prior to H locus rearrangement, Eμ-myc expression commences very early in B cell development (19). Accordingly, young Eμ-myc mice contained an abnormally expanded population of large B cell precursors and B cell differentiation was blocked at the Pre-B cell stage (31). In contrast, in λ-MYC mice MYC expression is driven by elements of the light chain and thus it would be expected to occur at a later stage, and not affect Pro-B cell subset. However, we found that in λ-MYC mice, transgenic MYC was expressed very early, with similarly elevated levels in Pro-B and Pre-B cells, suggesting that the Ig λ regulatory sequences included in the transgene are not sufficient to recapitulate the endogenous expression of the Ig light chain allele.

Notably, endogenous *c-Myc* expression was sharply reduced at the Pre-B cell stage in control mice. This is in agreement with the finding that, under normal conditions, *c-Myc* mRNA is detected during the maturation and expansion of Pro-B cells into Pre-B cells and is later only detectable in a fraction of mature B cells (32). Likewise, in a reporter model of endogenous c-Myc expression, GFP-c-Myc was only detectable in a fraction of Pro-B cells and was undetectable at subsequent differentiation stages (33). Downregulation of endogenous c-Myc could be due in part to repression of c-Myc expression by the pre-BCR (34). The failure of Pre-B cells in λ-MYC mice to downregulate *c-MYC* expression could explain the pro-apoptotic effect in this population and would indicate that downregulation of c-MYC expression is concomitant with differentiation of mature B cells. Thus, deregulated *c-MYC* expression in BM B cells results in a block at the Pro-B cell stage, associated with reduced survival and cell proliferation of Pre-B cells.

B cells require successful rearrangement of the Ig H and L chain gene loci and surface expression of the BCR in order to leave the BM and migrate to the periphery, where they undergo their final maturation steps in the spleen (35). However, B cells lacking surface BCR (Ig-) were overrepresented in the secondary lymphoid organs of pre-tumor stage λ-MYC mice. Interestingly, a large proportion of these cells expressed the GL7 marker, which defines distinct subsets of early differentiating B cells in the BM (28). GL7 is expressed in early proliferating B cells (28) and CD43 is expressed very early in differentiating B cells and is downregulated from the Pre-B cell stage (27, 36). Thus, we propose that B220+Ig-GL7+CD43- spleen cells represent a Pro-B/Pre-B population that is able to escape the BM and populate the secondary lymphoid organs of pre-tumor stage λ-MYC mice. Consistent with this idea, these cells are highly proliferative.

Independent studies of the role of AID in MYC-driven lymphomagenesis in Eμ-Myc mice have produced variegated results (37, 38), which could possibly be explained because the Eμ-Myc model is a model of pre-B/immature lymphoma (19), with mature B cell lymphomas accounting for only 19% of tumors. By using the λ-MYC Tom^+/ki^ model, we provide a more granular picture of c-MYC-driven B cell lymphomagenesis, and a characterization of λ-MYC mouse model that differs considerably from the original study (20). Our analysis reveals that Tom+ (AID-experienced) cells make only a minor contribution to tumors in these mice and that B cell lymphomas generated in the λ-MYC model originate from B cells at various maturation stages, with only a small fraction of them resembling BL. In the original study, tumors were described as IgM+ and pathologically resembled BL, although the analysis lacked full phenotypic characterization of tumors (20). In a later study, tumors in λ-MYC mice were defined as pre-GC (17). Our study shows that a large fraction of tumors in λ-MYC mice are immature and IgM-. Moreover, the use of Tomato fluorescent protein as a reporter of AID expression allowed us to precisely detect AID-experienced IgM+ tumors, revealing a complexity of mature tumors that would otherwise have passed unnoticed. Our results show that only a minor fraction of the tumors generated in λ-MYC mice recapitulate key features of the Burkitt pathology in humans.

The lower aggressiveness of tumors containing Tom+ IgM+ cells suggests that AID expression defines a distinct pathological entity, with lower aggressiveness than less mature Tom- tumors and Ig- tumors. This idea is supported by the differential pathway regulation between Tom+ and Tom- IgM+ cells. For example, the RNA-seq analysis detected elevated apoptosis in Tom+ tumors. In addition, pathway enrichment analysis showed that PI3K signaling pathway was significantly active in Tom+ cells, accompanied by inhibition of PAK signaling. PI3K signaling is a major proliferation and survival pathway deregulated in BL (29). PAK (p21 activated kinase) signaling has been previously described as a mechanism for promoting cell proliferation and cell invasion (39). In addition, PAK1 has been shown to be involved apoptosis inhibition (40).

In support of this notion, GSEA analysis of a gene set downregulated in GC/post-GC tumors (17) from λ-MYC/IμHABCL6 mice (41) revealed a strong association with the expression pattern found in Tom+ IgM+ tumors, whereas Tom- IgM+ cells were more similar to pre-GC tumors from λ-MYC mice. This reinforces the notion that λ-MYC mice generate different subtypes of B cell neoplasias. Further, our results indicate that AID-experienced lymphoma B cells have a distinct transcriptional identity typical of GC-derived subtypes of B cell lymphoma

AID-induced mutations and translocations are known to contribute to B cell lymphomagenesis (14, 15, 42, 43). In addition, AID expression has been linked to poor prognosis in hematological cancers (17, 44, 45). Thus, our finding of that AID expression is a positive prognostic indicator in λ-MYC tumors is somewhat unexpected. This apparent contradiction could be explained by the etiology of the different tumor subtypes, i.e. immature B cell lymphomas are typically more aggressive than mature (AID-experienced) B cell lymphomas. One alternative explanation is that AID expression could increase the burden of non-synonymous mutations, resulting in the appearance of tumor neoantigens (neoAgs). NeoAg load has been proposed as a biomarker in cancer immunotherapy (46), as they could increase the immunogenicity of the tumor and favor its elimination (47, 48). Cancer immunotherapies, such as adoptive T cell therapy, personalized cancer vaccines, and immune checkpoint inhibition, in turn rely on understanding patient-specific neoAgs (49, 50). Recent studies have shown that AID-driven mutations can reduce B cell fitness (51) and that AID mutational load independently associated with a favorable outcome in immune-checkpoint inhibitors treated patients (52). The idea that AID could contribute to neoantigen generation and immunogenicity is thus an attractive hypothesis which deserves further investigation.

## Data availability statement

The datasets generated for this study can be found in the Gene Expression Omnibus repository (GSE239433).

## Ethics statement

All animal procedures conformed to EU Directive 2010/63EU and Recommendation 2007/526/EC regarding the protection of animals used for experimental and other scientific purposes, enacted in Spanish law under RD 53/2013. The procedures were reviewed by the CNIC Institutional Animal Care and Use Committee and approved by the Community of Madrid. The study was conducted in accordance with the local legislation and institutional requirements.

## Author contributions

CG-E: Conceptualization, Writing – original draft, Formal Analysis, Investigation, Methodology, Validation. EM-Z: Formal Analysis, Investigation, Methodology, Writing – review & editing. AR: Conceptualization, Funding acquisition, Project administration, Supervision, Writing – original draft.
