# Supplementary material for: Activation-induced deaminase expression defines mature B cell lymphoma in the mouse
Source: Front Immunol. 2023 Sep 21;14:1268930. doi: 10.3389/fimmu.2023.1268930 (PMC10558245; doi:10.3389/fimmu.2023.1268930)
Supplement: Supplementary file 1 [file DataSheet_1.docx]

Supplementary Material

Activation-induced deaminase defines a mature Burkitt-like lymphoma in the mouse

Carmen Gómez-Escolar^1^, Ester Marina-Zárate^1,2^, Almudena R. Ramiro^1^

^1^ B Lymphocyte Biology Lab. Centro Nacional de Investigaciones Cardiovasculares (CNIC). Madrid, Spain

^2^Current address: La Jolla Institute for Allergy & Immunology. CA, US.

*** Correspondence:** Corresponding Author: aramiro@cnic.es

# Supplementary Figures and Tables

## Supplementary Figure 1


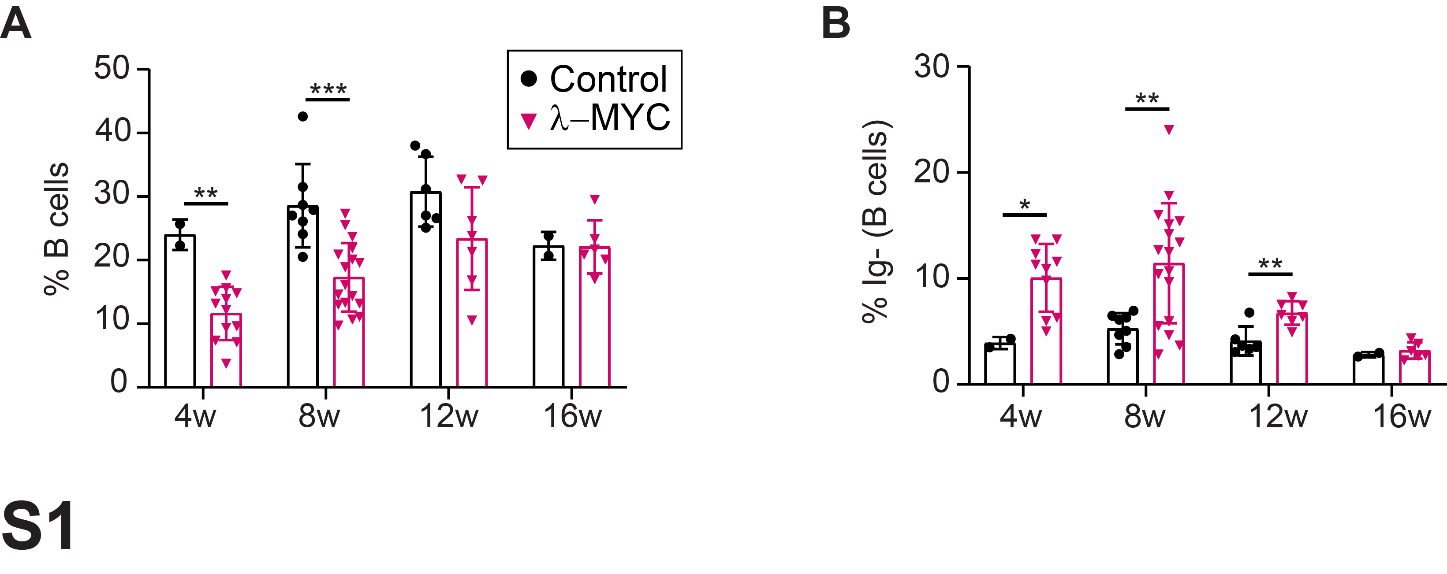


**Supplementary Figure 1. B cell lymphopenia in lymph nodes of pre-tumor stage λ-MYC mice.** (A, B) Quantification of B cells (B220+) (A) and Ig- B cells (B) in lymph nodes from λ-Myc+/+ (control) and λ-Myc+/TG (λ-MYC) mice. Data were analyzed by unpaired t test. *P<0.05, **P<0.01, and ***P<0.001.

## Supplementary Figure 2


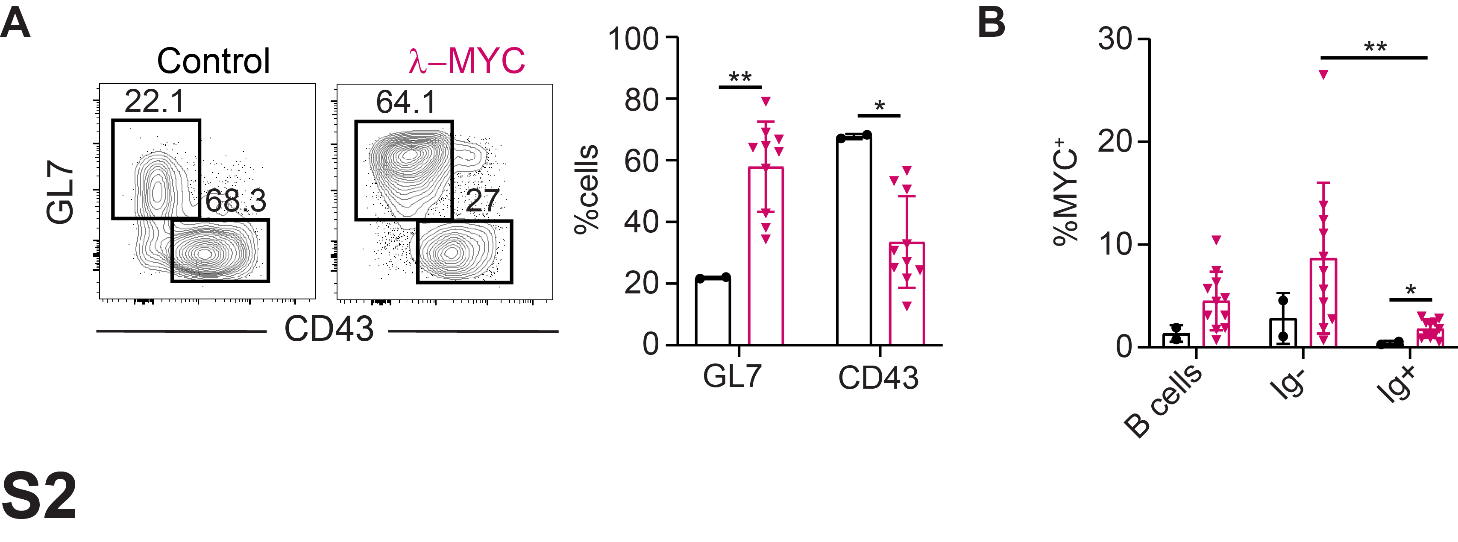


**Supplementary Figure 2. Characterization of Ig- cells in secondary lymphoid organs of pre-tumor stage λ-MYC mice (A)** Representative flow cytometry plots and quantification of GL7 and CD43 expression in Ig- splenic B cells from 8-week-old control and λ-MYC mice. (B) Quantification of MYC+ cells as a percentage of the total B cell, Ig+ B cell, and Ig- B cell populations in the lymph nodes of 8-week-old control and λ-MYC mice. Data were analyzed by unpaired t test. *P<0.05 and **P<0.01.

## Supplementary Figure 3


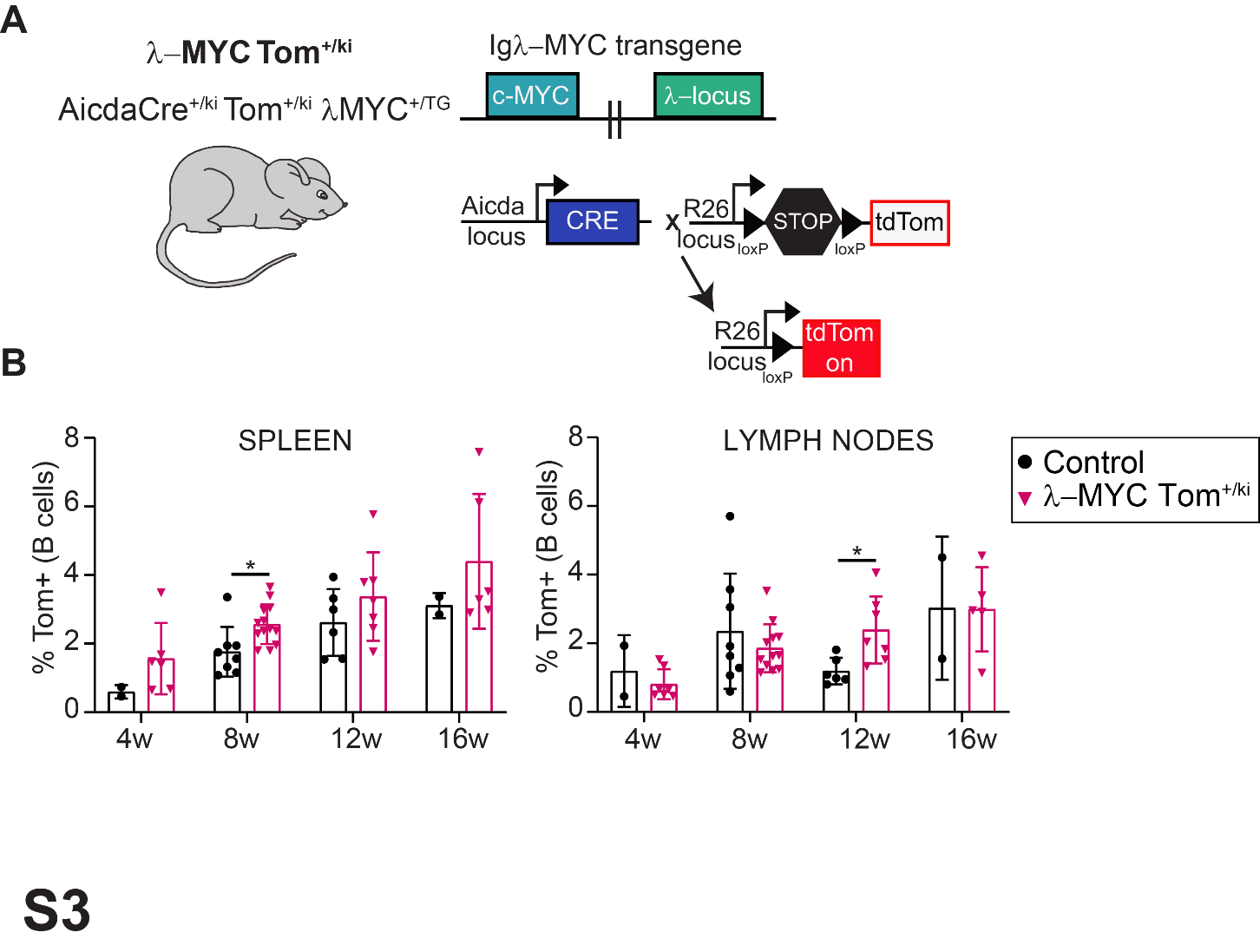


**Supplementary Figure 3. AID-experienced (Tom+) cells are detectable in the secondary lymphoid organs of pre-tumor stage λ-MYC Tom^+/ki^ mice. (A)** Genetic approach used to monitor AID-experienced cells during MYC-driven lymphomagenesis. In *AicdaCre^+/ki^Tom^+/ki^λ-MYC^+/TG^* mice (λ-MYC Tom^+/ki^), expression of endogenous AID drives expression of Cre recombinase, which in turn activates expression of the Tom fluorescent protein. **(B)** Quantification of the proportion of B cells expressing Tom fluorescent protein in the spleen and lymph nodes of control and λ-MYC Tom^+/ki^ mice. Data were analyzed by unpaired t test. *P<0.05.
